# mRNA vaccine for cancer immunotherapy

**DOI:** 10.1186/s12943-021-01335-5

**Published:** 2021-02-25

**Authors:** Lei Miao, Yu Zhang, Leaf Huang

**Affiliations:** Division of Pharmacoengineering and Molecular Pharmaceutics, Eshelman School of Pharmacy, University of North Carolina at Chapel Hill, Chapel Hill, NC 27599 USA

**Keywords:** Self-amplifying mRNA (SAM), mRNA delivery, Ionizable lipids, Lipid nanoparticles (LNPs), Cancer vaccine, Cancer immunotherapy, Personalized vaccine

## Abstract

mRNA vaccines have become a promising platform for cancer immunotherapy. During vaccination, naked or vehicle loaded mRNA vaccines efficiently express tumor antigens in antigen-presenting cells (APCs), facilitate APC activation and innate/adaptive immune stimulation. mRNA cancer vaccine precedes other conventional vaccine platforms due to high potency, safe administration, rapid development potentials, and cost-effective manufacturing. However, mRNA vaccine applications have been limited by instability, innate immunogenicity, and inefficient in vivo delivery. Appropriate mRNA structure modifications (i.e., codon optimizations, nucleotide modifications, self-amplifying mRNAs, etc.) and formulation methods (i.e., lipid nanoparticles (LNPs), polymers, peptides, etc.) have been investigated to overcome these issues. Tuning the administration routes and co-delivery of multiple mRNA vaccines with other immunotherapeutic agents (e.g., checkpoint inhibitors) have further boosted the host anti-tumor immunity and increased the likelihood of tumor cell eradication. With the recent U.S. Food and Drug Administration (FDA) approvals of LNP-loaded mRNA vaccines for the prevention of COVID-19 and the promising therapeutic outcomes of mRNA cancer vaccines achieved in several clinical trials against multiple aggressive solid tumors, we envision the rapid advancing of mRNA vaccines for cancer immunotherapy in the near future. This review provides a detailed overview of the recent progress and existing challenges of mRNA cancer vaccines and future considerations of applying mRNA vaccine for cancer immunotherapies.

## Introduction

Cancer immunotherapies have gained tremendous attention since the U.S. Food and Drug Administration (FDA) approval of six checkpoint blockade modulators and two chimeric antigen receptor (CAR)-T cell immunotherapies [1, 2]. Cancer immunotherapies aim to activate the host anti-tumor immunity, modify the suppressive tumor microenvironment and ultimately result in tumor reduction and increased overall patients’ survival rate. Cancer vaccines are an attractive alternative immunotherapeutic option with both prophylactic and therapeutic potentials. The vaccines that target tumor-associated or tumor-specific antigens (TAAs or TSAs) can specifically attack and destroy malignant cells that overexpress the antigens and achieve chronic therapeutic response because of immunologic memory. Therefore, cancer vaccines offer specific, safe, and tolerable treatment compared to other immunotherapies. Despite considerable efforts to develop cancer vaccines, clinical translations of cancer vaccines into efficacious therapies have remained challenging for decades due to highly variate tumor antigens and relevantly low immune response. Nonetheless, U.S. FDA has recently approved two prophylactic vaccines, one for human papillomavirus (HPV) that accounts for 70% of cervical cancers, and another for hepatitis B virus that can cause liver cancer [3]. More encouragingly, PROVENGE (Sipuleucel-T), an immune cell-based vaccine has been approved by the U.S. FDA in 2010 as the first therapeutic cancer vaccine for treating hormone-refractory prostate cancer patients [4]. Besides these initial successful attempts in cancer vaccines, multiple personalized cancer vaccines combined with checkpoint blockage modulators or cytokine therapies are currently being evaluated in clinical trials, with some promising clinical responses in multiple solid or metastatic tumors [5, 6].

There are four types of cancer vaccines, including tumor or immune cell-based vaccines, peptide-based vaccines, viral vector-based vaccines, and nucleic acid-based vaccines [7]. Nucleic acid (DNA- or RNA-) based vaccine is a promising vaccine platform for multiple reasons. Firstly, nucleic acid vaccines allow simultaneous delivery of multiple antigens covering various TAAs or somatic tumor mutations, eliciting both humoral and cell-mediated immune response, increasing the likelihood of overcoming vaccine resistance. Secondly, unlike peptide vaccines, nucleic acid vaccines can encode full-length tumor antigens, allowing APCs to simultaneously present or cross-present multiple epitopes with both class I and II patient-specific human leukocyte antigen (HLA), thus are less restricted by the human HLA types and more likely to stimulate a broader T cell response [8]. Ultimately, nucleic acid vaccines are non-infectious, free of protein or virus-derived contaminations during production, and are thus considered well tolerated for both prophylactic and therapeutic applications [7]. Messenger RNA (mRNA) vaccine has recently emerged as an appealing alternative to DNA vaccine for infectious disease preventions and anti-cancer treatments. Advantages of mRNA over DNA as cancer vaccine strategy include: (1) mRNAs can be translated in both dividing and non-dividing cells, where RNA only needs to be internalized into the cytoplasm, followed by a one-step translation into the antigen(s) of interest. The rate and magnitude of protein expression of mRNA are typically higher than DNA vaccines. (2) Unlike DNA vaccines, mRNA vaccines cannot integrate into the genome sequence, thus free of insertional mutagenesis. The first report of the successful expression of in vitro transcription (IVT) mRNA in mouse skeletal muscle cells through direct injection into animals was published in 1990, underlining the feasibility of mRNA vaccine development [9]. However, this early attempt didn’t lead to substantial mRNA vaccine development investigations, largely stemmed from concerns regarding mRNA instability, insufficient in vivo delivery, and high intrinsic innate immunogenicity [10].

Over the past decades, major technological innovations have enabled mRNA as a more feasible vaccine candidate. Various modifications of mRNA backbone and untranslated regions make mRNA less RNase-sensitive, more stable, and highly translatable. Improved purification methods have allowed mRNA products free of double-stranded contaminations, thus reducing the non-specific activation of innate immunity. More efficient in vivo delivery of mRNA has been achieved by formulating mRNA into delivery vehicles, including but not limited to lipid nanoparticles (LNPs), polymers, and peptides. Lastly, IVT methods (free from isolation and purification of biological samples) have been widely applied to the production of mRNAs. With the maturation of scale-up manufacturing, mRNA vaccines have supreme advantages over other vaccine techniques due to the rapid, inexpensive production and large-scale deployment [11]. So far, non-replicating mRNAs are mostly investigated in clinical trials for cancer treatment. However, self-amplifying mRNAs (SAM) have gained extensive attention and are being evaluated in both cancer and infectious disease due to long-lasting efficacy and lower required dosages [12, 13].

Up to now, over twenty mRNA-based immunotherapies have entered clinical trials with some promising outcomes in solid tumor treatments. Besides anti-cancer immunotherapies, mRNA vaccines have a vast advantage to respond rapidly to the global explosion of the coronavirus disease 2019 (COVID-19). With the recent U.S. FDA’s approval of two mRNA-based vaccines from Pfizer-BioNTech and Moderna for emergency use in COVID-19 prevention, the mRNA vaccine field will encompass a dramatic rise in the market value and will attract widespread interest in both cancer and infectious disease applications [14, 15]. In this review, we discuss the improvements that have been made on mRNA structures to increase stabilities and translation efficiencies, highlight the advantages and limitations of various in vivo delivery vehicles for mRNA therapeutics, evaluate the applications of SAM in cancer vaccines, and summarize the current clinical applications of mRNA cancer vaccines. The data suggest mRNA vaccines have the potential to overcome several challenges for cancer immunotherapies.

## Basic mRNA pharmacology, limitations and advantages

mRNA is a single-stranded macromolecule that corresponds to the genetic sequence of a DNA in the cell nuclei and is read by a ribosome and translated into proteins in the cytoplasm [16]. The rationale behind mRNA as an appealing cancer vaccination platform is to deliver the transcript of interest(s), encoding one or more TAAs or TSAs, into the host cell (typically APCs) cytoplasm, to be expressed into the targeted antigen(s). The expressed TAAs and TSAs can be presented to the surface of APCs by major histocompatibility complexes (MHCs) to activate anti-tumor immunity. mRNA vaccine could induce both antibody/B cell mediated humoral responses and CD4^+^ T/ CD8^+^ cytotoxic T cell responses, which are beneficial for efficient clearance of malignant cells. On the other side, mRNA is non-infectious and non-integrating, and therefore it’s quite tolerable and has posed no genetic risks. There are mainly three types of RNAs currently investigated as cancer vaccines: non-replicating unmodified mRNA, modified mRNA and virus derived SAM. IVT has been commonly used for synthesizing both non-replicating mRNA (modified and unmodified) and SAMs. The method utilizes a bacteriophage RNA polymerase, such as T3, T7 or SP6 RNA polymerase and a linearized DNA template containing the target antigen sequences. The IVT production precludes the usage of cells and their associated regulatory hurdles, and therefore the production of mRNA is undoubtedly simpler, quicker and cleaner than large-scale protein production and purification. The fundamental structure of conventional non-replicating IVT mRNA, which correspondent to “mature” eukaryotic mRNA, is composed of an open reading frame (ORF) region that encodes the target antigen sequences, flanked by five-prime (5′) and three-prime (3′) untranslated region (UTR), and further stabilized by 7-methylgaunosine (m7G) 5′ cap and 3′ poly (A) tails respectively. The 5′ cap and 3′ poly (A) can be added during the IVT or added enzymatically after initial IVT. In contrast, SAM comprises two ORFs, including one that encodes the targeted antigen sequences and another that encodes viral replication machinery which enables long-lasting RNA amplification intracellularly. Once mRNA or SAM is internalized and transited to the cytosol, it will be read by ribosomes, and translated into proteins that undergoes post-translational modifications, ultimately resulting in a properly folded functional protein. The remaining IVT mRNA template will be degraded by normal physiological process, decreasing the metabolite toxicity risk [11].

There are several limitations for initial applications of mRNA in vaccine development. First, naked mRNA is quickly degraded by extracellular RNases, and is not internalized by APCs efficiently. Secondly, mRNA has intrinsic immunogenicity, which can activate downstream interferon related pathway to elicit innate immunity. Although this intrinsic immunogenicity can be functioned as adjuvant-like effect to boost immune response, however, it paradoxically facilitates mRNA degradation, reducing antigen expression. Moreover, the impurities, mainly double stranded RNA (dsRNA) generated during IVT process, will potentiate the activation of innate immunity, further limiting mRNA translation. In the following sections, we will discuss these limitations and strategies to overcome these limitations.

## Immunogenicity of mRNA and paradoxical effects in Cancer immunotherapy

Innate immune response is usually activated by host immune system through detecting exogeneous motifs called pathogen-associated molecular patterns (PAMPs) via the pattern recognition receptors (PRRs) [17]. These receptors are particularly highly expressed in APCs, the major target cell population of mRNA cancer vaccines. Exogeneous IVT mRNA is intrinsically immunostimulatory, as it is recognized by a variety of cell surface, endosome and cytosolic PRRs [11]. Recognition of IVT mRNA inside the endosome is mainly mediated by toll-like receptor (TLR)-7 and − 8 (one type of PRRs), subsequently activates the myeloid differentiation marker 88 (MyD88) pathway, leading to activation of Type-1 interferon (IFN) pathways and secretion of proinflammatory cytokines [17, 18]. In the cytosol, these exogeneous mRNAs are sensed by other PRR families, including retinoic acid-inducible gene-I-like (RIG-I-like) receptors, oligoadenylate synthetase (OAS) receptors, and RNA-dependent protein kinase (PKR). These PRRs can sense different RNAs, including dsRNA and single stranded RNA (ssRNA), blocking mRNA translation as reviewed elsewhere [17].

The activation of multiple PRRs and production of type I IFN can be paradoxically beneficial or detrimental for anti-cancer immunotherapy. It is potentially beneficial for vaccination since, in some cases, activation of type I IFN pathways drives APC activation and maturation, promotes antigen presentation, and elicits robust adaptive immune responses. However, innate immune sensing of RNAs may be associated with inhibition of antigen expression, and thus dampen immune response. Specifically, phage RNA polymerases produce unwanted dsRNA during IVT that can activate innate immunity via PKR, OAS, TLR-3, MDA-5 (one type of RIG-I like receptors). Once the PKR is activated, the eukaryotic initiation factor (eIF)-2 can be phosphorylated, blocking mRNA translation [17]. Moreover, the dsRNA activates RNase L upon binding to OAS [19], causing degradation of the exogenous RNAs. Ultimately, binding of dsRNA with MDA-5 and TLR-3 can activate Type I IFN, eliciting several other genes that inhibit the translation of mRNA [20]. Besides the dsRNA impurities, improperly designed mRNA structure may also activate PRRs like MDA-5 and PKR, abolishing antigen expression.

The paradoxical impact of Type I IFNs activation is not only restricted to antigen expression, but also shown on CD^+^ 8 T cell activation. The dual effect of Type I IFNs on CD8^+^ T cell immunity have been extensively reviewed elsewhere [21]. In brief, the stimulatory or inhibitory actions of type I IFNs on CD^+^ 8 T cell activation is likely to be dependent on the timing and kinetics between activation of IFNAR signaling and TCR signaling, which may be further impacted by the routes of administration of mRNA cancer vaccines. For instance, several studies have shown that type I IFNs can potentially promote CD8^+^ T cell response to systemic mRNA vaccination [18, 22]. One hypothesis is that, intravenous (i.v.) delivery of mRNA (typically delivered by cationic lipoplex) is expressed in splenic DCs [18, 22], where antigen expression and presentation take place simultaneously, with TCR signaling preceding or coinciding with IFNAR signaling. In contrast, type I IFNs can potentially interfere with topical (i.d. or s.c.) mRNA vaccination where antigen expression happens locally in the injection site, but antigen presentation takes place in the secondary lymphoid organs, with IFNAR signaling precedes TCR signaling [23, 24]. However, this IFNAR/TCR signaling theory is still debating, since other research groups have observed the opposite effects from local administration of mRNA vaccines [25, 26]. Therefore, the purity of mRNA products, the modification of mRNA sequence, the design of delivery system and administration routes need to be tuned to properly active the innate immunity to initiate the adaptive immune response, simultaneously, averting the toxic overactivations that inhibit antigen protein expression and immune response.

## Strategies to improve mRNA translation efficiency and overcome the innate immunogenicity

### Five-prime cap (5’Cap) modification

IVT mRNAs, which mimic the eukaryotic mRNA, usually have a N7-methylated guanosine added to the first 5′ nucleotide through a 5′, 5′-triphosphate bridge for efficient translation in the eukaryotic system. This 5′ m7G cap or m7Gppp- is typically referred to as “Cap 0”. The 5′ cap recruits the eukaryotic translation initiation factor 4E (eIF4E) to facilitate ribosome recognition and translation initiation. Both enzymatic and chemical strategies are applied for mRNA 5′ capping. The most widely used in vitro post-translational capping enzymatic method is the Vaccinia capping system, which is based on the Vaccinia virus Capping Enzyme (VCE) [27]. The VCE consists of 2 subunits (D1 and D12). The D1 subunit possesses triphosphatase, guanylyltransferase, and methyltransferase activity, all of which are essential for adding a complete Cap 0 structure, while D12 plays a valid role in activating D1 [28]. Vaccinia capping system provides a near 100% capping efficiency with proper orientation, but efficient expression and purification for VCE are required for large scale capped RNA production [29]. Besides the enzymatic post-translational capping methods, chemical capping methods add cap analogs co-transcriptionally. However, regular cap analog added during IVT (co-transcriptional process) can be reversely incorporated into the mRNA sequence. Therefore, approximately one third of mRNA molecules are not properly methylated, with free phosphate hanging at the 5′ location, leading to low efficiency of downstream mRNA translation. To prevent reverse incorporation, anti-reverse cap analogs (ARCA) have been developed. ARCA is methylated at the C3 position (closer to m7G) to ensure the addition of a nucleotide only at the non-methylated guanosine during IVT. ARCA capped mRNA increases and prolongs protein expression in vitro. To inhibit de-capping of the corresponding mRNA and increase binding affinity to eIF4E, ARCA have been further modified within the triphosphate linkage, either through a bridging oxygen (e.g. (methylenebis) phosphonate and imidodiphosphate) or a non-bridging oxygen (e.g. phosphorothioate and phosphorselenoate) [30, 31]. Remaining limitations of ARCA caps are: (1) Relatively low capping efficiency (60–80%); (2) Cap-0 structure is formed after capping; (3) Cap contains an unnatural O’methyl group in the C3 position that can be recognized as exogeneous motif; (4) mRNA transcript must start with guanine (G). 5’cap can be added enzymatically after IVT to achieve 100% capping efficiency with a natural unmodified cap structure. However, the process is costly and suffers from batch to batch variability. A next generation co-transcriptional cap analog, CleanCap™, was developed in 2018 to overcome the issues associated with ARCA [32]. CleanCap™ utilized an initiating capped trimer to yield a natural unmodified cap structure with increased capping efficiency to nearly 90–99%.

Uncapped (5’ppp or 5’pp) or abnormally capped (Cap-0) mRNAs can be recognized by PRRs [33], such RIG-1 and IFIT, triggering type I IFN, blocking mRNA translation [20, 34, 35]. Therefore, a natural Cap-1 structure is preferred. Cap1 structure can be enzymatically added by guanylyl transferase and 2′-O-methyltransferases or through the co-transcriptional CleanCap™ technology. To further avoid recognition by the innate immune system, capped-IVT mRNAs should be treated with phosphatases to remove uncapped phosphate, preventing PRR-mediated sensing and destruction of mRNA translation [36].

### Optimization of Untranslated regions (UTRs)

UTRs can impact mRNA degradation rate and translation efficiency through interacting with RNA binding proteins. 5′ UTR sequence can be optimized to enhance the stability of mRNA and accuracy of translation. Firstly, avoid the presence of start codon (AUG), and non-canonical start codons (CUG) in the 5′ UTR, as these codons may disturb the normal translation process of ORF. Secondly, avoid the presence of highly stable secondary structures, which can prevent ribosome recruitment and codon recognition. Thirdly, shorter 5’UTR may be introduced as previous studies have shown that this type of 5’UTR is more conducive to mRNA translation process. Ultimately, bioinformatics tool can be used to predict mRNA translation efficiency according to 5’UTR sequence. α-globin and β-globin from *Xenopus laevis* or humans contain translation and stability regulatory elements, and are commonly used as the 3′ UTR of IVT mRNA [37]. To further improve RNA stability, AU- and GU-enriched sequences can be introduced. Moreover, transcription efficiency might be improved by adding 3’UTR sequence twice in tandem [38]. Overall, UTR performance is dependent on species, cell type, and cell state. One needs to understand the pharmacology in the targeted cells to allow better design of UTRs of the therapeutic mRNA vaccines [39].

### Codon optimization of open Reading frame (ORF)

Optimization of G and cytosine (C) content in the ORF can be applied to regulate the translation elongation rate. Uridine depletion is another codon optimization strategy that can directly be linked to an increased GC content. Uridine-rich regions can be recognized by RIG-I, and its activation may lead to abolishing of protein expression. Moreover, the sequence can be optimized to have the same ratio of every codons found naturally in highly expressed proteins in the targeted cells or to use the best pairs of codons that are commonly seen in these highly expressed proteins. In addition, codons with higher tRNA abundance are usually used to replace rare codons in ORF to increase the translation rate. Lastly, highly stable secondary structures and hairpin loops should be avoided in the ORF [17]. However, high translation rate is not all beneficial, as some proteins require a low translation rate to correctly and effectively fold [31]. Therefore, codon optimizations in the ORF should be carefully monitored to ensure moderate translation rate and high translation accuracy. Thess et al. demonstrated that sequence engineered but chemical unmodified mRNA is fully suited for use in mRNA therapies, and the protein expression level was even higher than chemically modified but without codon optimized mRNA, indicating the importance of codon optimization in improving mRNA expression efficiency [40].

### Poly (a) tail modification

Poly(A) sequence can slow down the degradation process of RNA exonuclease, increase RNA stability, and enhance translation efficiency. A suitable length of Poly(A) is crucial. Commonly used Poly(A) is 250 units in length, but different cells may have different preferences. For example, the optimal length of poly(A) in human monocyte-derived DCs are 120–150 nucleotides, in human primary T cells are 300 nucleotides [17]. Moreover, Poly (A) binding protein (PABP) can interact with 5’cap through translational initiation factors, such as eIF4G and eIF4E, forming a close-loop to impact mRNA structure [17, 41]. Recent study by Lima and coworkers found that shorter poly(A) sequence could promote this closed-loop structure for efficient translation [41]. Therefore, future studies should evaluate the role of poly-A size in kinetic expression of IVT-mRNA antigen [17, 21].

### Nucleoside modified mRNA

Another method to improve mRNA stability, translation efficiency and mRNA vaccine potency is to modify mRNA transcripts with alternative nucleotides [42–45]. Pseudouridine (Ψ), 1-methylpseudouridine (m1Ψ), and 5-methylcytidine (m5C) are used to replace the natural uridine and cytidine, and thus to remove intracellular signaling triggers for PKR and RIG-I, leading to enhanced antigen expression. Kariko et al. have found that altering nucleosides in the mRNA’s structure (e.g., 5mC or Ψ) can substantially reduce innate immune activation and increase translational capacity of mRNA [44, 46–48].

Post-transcriptional epigenomic RNA modifications can also be a powerful approach for improving mRNA translation and evading innate immune response. Arango and coworkers reported that post-transcriptional RNA modification with N4-aceylcytidine (ac4C) enhanced mRNA translation in vitro and in vivo [49]. Moreover, the function of post-translational epigenomic modifications in DC activation has been demonstrated by mettl3, an RNA methyl transferase which mediates mRNA m6A methylation and induces DC activation [17, 50].

### Purification of IVT-mRNA

As mentioned in section 2, phage polymerase in IVT can yield multiple contaminants, including short RNAs generated from abortive initiation event and dsRNA produced by self-complementary 3′ extension [46]. These RNA contaminants can activate intracellular PPRs, including PKR, MDA-5, OAS etc. and lead to abolish of mRNA translation and activation of innate immunity. Kariko and coworkers have demonstrated that the removal of these RNA contaminants result in mRNA that does not induce IFNs and inflammatory cytokines, ultimately leading to10- to 1000-fold increase in protein production in human primary DCs [46]. dsRNA species can be reduced during IVT by decreasing Mg^2+^ concentration or by producing RNA at elevated temperature [17]. A more complete and scalable removal of dsRNA was performed by high-pressure liquid chromatography (HPLC) [46, 51]. However, HPLC purification of mRNA is usually high cost and low yield (< 50%). Recently, a fast and cheap purification method has been reported by Baiersdorfer et al. The method utilized the selective binding of dsRNA to a cellulose powder in ethanol containing buffer combined with fast protein liquid chromatography (FPLC) to remove up to 90% of dsRNA [52]. Another way to completely get rid of dsRNA contaminants is through solid phase synthesis of mRNA rather than IVT. For instance, Shivalingam et al. has synthesized RNA fragments up to ~ 70 nucleotides using the solid phase method. The RNA fragments were then ligated to become full length mRNA. This process is scalable and completely avoids the formation of dsRNA [53].

### Utilizing the impact of type I IFN for improved mRNA vaccination

As mentioned earlier, type I IFN shows paradoxical impact on the immune response of mRNA cancer vaccine. Several studies have demonstrated that increased innate immune stimulation driven by mRNA and delivery system modifications are not necessary for increased immunogenicity [23, 24, 54]. Other studies indicated that enhanced immune responses via combination with alternative adjuvants are required for mRNA vaccines to achieve the targeted anti-tumor therapeutic outcome and improved patients’ survival. Islam and coworkers have reported mRNA pulsed with a palmitic acid-modified TLR7/8 agonist R484 markedly improved the MHC class I presentation of OVA mRNA derived antigen in APCs, subsequently induced a more effective adaptive immune response in a tumor bearing mouse model as compared to mRNA vaccine without the adjuvant [55]. Moreover, the RNActive® vaccine platform developed by CureVac AG. used RNA/protamine complex as an adjuvant to activate TLR7/8, induce Th1 T cell response. Enhanced antitumor immunity was achieved when dosing RNA/protamine adjuvant with the naked, unmodified mRNA encoding antigens [5]. In addition to using TLR agonists as adjuvants, stimulator of interferon genes (STING) agonists have been recently applied as immunomodulators for combination with mRNA and peptide vaccines [56, 57]. Miao et al. have shown that loading of mRNA cancer vaccines into LNPs with intrinsic STING-IFN activation function produced a potent and prolonged CD8^+^ T cells response [57]. Improved antitumor efficacies were observed in three cancer models with the addition of STING activating lipids. Recently, a combination of pro-inflammatory cytokines and chemokines have also been exploited to boost the antitumor immunity of mRNA vaccines in both pre-clinical and clinical studies. In one clinical study, a DC-based mRNA vaccination composed of a mixture of TAAs were administrated together with DCs electroporated with mRNA encoding CD70, CD40 ligand (CD40L), and constitutively active TLR4 (TriMix). The combination therapy resulted in an encouraging rate of tumor responses in patients with stage III or IV melanoma [58]. Costimulatory molecules CD70 and CD40L, together with active TLR4, play crucial roles in the activation of DCs and priming of CD8^+^ T cell responses [59]. The cytokine cocktails are not only used to prime DC and T cell functions, but can also be dosed intratumorally to reshape the tumor microenvironments. For instance, intratumoral injection of mRNA-encoding cytokines IL-23, IL-36Ƴ, and T cell co-stimulatory OX40L can overcome the suppressive tumor environment and produce effective systemic antitumor immunity [60]. Studies in combination of adjuvants with mRNA vaccines are blooming, but this strategy should be used with caution as it could be counterproductive and paradoxical, especially when using immune-stimulatory molecules that have tight interactions with type I IFN and the innate immunity pathway.

## Self-amplifying mRNA vaccine, structure, advantages and deliveries

Another RNA vaccine platform that holds the promise to maximize the magnitude and length of antigen production is SAM. SAMs are originated from positive-single stranded mRNA viruses, most commonly from alphaviruses such as Sindbis and Semliki-Forest viruses [13]. The structural protein encoding genes of respective alphavirus that enable the formation of infectious viral particles have been replaced with gene encoding the antigen(s) of interest, whereas the RNA replication machinery remains. Specifically, the viral RNA-dependent RNA polymerase (known as replicase) and the non-structural proteins were retained to assemble into the multi-enzyme replicase complex to direct cytoplasmic amplification of RNA [61]. SAM can self-amplify over time (up to 2 months) and consequently inducing more potent and persistent immune responses owing to the integrity of the viral replication machinery. The SAM platform precedes other nonreplicating mRNA vaccine platforms in that it allows for a huge amount of antigen production in an extended period of time from a remarkable low dose vaccination [11]. Johanning et al. reported that the i.m. injection of Sindbis virus-derived SAM led to a ten-fold increase in antigen expression ratio and eight-day extension of expression (from 2 to 10 days) than non-replicating mRNA [62].

Early investigation of SAM is direct injection of SAM packaged into viral replication particles (VRP) [63, 64]. VRPs are potent vaccines in mice [65], non-human primates and humans [66]. However, the replicated VRP structural proteins may induce non-specific immunogenicity and toxicity. To decrease the infectious concern of viral components, a propagation-defective type of VRPs was generated. The capsid and envelope proteins of the modified VRP are encoded in trans as defective helper constructs during production. Only the RNA can further amplify after internalization, whereas other part of VRPs lack the ability to form infectious viral particles [67]. Nowadays, complete synthetic SAM produced after IVT can be directly used as RNA-based vaccine, removing the potential safety concerns of the viral components. Since SAM is a huge and negatively charged molecule (~ 9500 nt), a delivery system is needed for its effective cellular uptake and protection from enzymatic degradation.

Over the past few years, substantial efforts have been made to identify a suitable delivery vehicle for IVT SAM. Medium-length cationic polymer polyethylenimine (PEI) was adopted by Vogel et al. to deliver the long SAM, from which they have shown that 64-fold less dose of SAM achieved the equivalent immunity to the non-replicating mRNA [68]. To decrease the potential toxicity from non-degradable cationic polymer, a bio-reducible, linear cationic polymer called “pABOL” was developed to deliver SAM. Blakney et al. demonstrated pABOL enhanced protein expression via both intramuscular (i.m.) and intradermal (i.d.) injection [69]. Geall and colleagues presented a new vaccine platform based on self-amplifying RNA encapsulated in synthetic LNPs. The LNP platform protected SAM from enzymatic degradation, allowed for efficient gene delivery after i.m. injection. Proof of concept was demonstrated in a model of respiratory syncytial virus (RSV) infection [13, 70]. To further improve transgene expression and immunity of SAM vaccines, several approaches have been attempted: Manara has reported the co-administration of GM-CSF expressing RNA with SAM to improve the potency against a lethal influenza virus challenge in mice [71]. Moreover, Lou et al. and Anderluzzi et al. both evaluated different cationic lipid formulations including liposomes, LNPs, polymeric nanoparticles and emulsions to encapsulate rabies virus glycoprotein G (SAM-RVG), and noticed that DOTAP containing polymeric nanoparticles and LNPs were the most potent in triggering humoral and cellular immunity [72, 73]. Lastly, SAM has been truncated into two transcripts (smaller in size) to address the concerns of inefficient delivery [74]. Beissert and the coworkers have separated SAM encoding the antigen of interest from the replicase activity. The replicase activity is provided in trans using a co-transfected RNA. These two compartment SAM demonstrated 10–100-fold higher transreplicon expression than the whole-set counterpart [74]. The doses of influenza hemagglutinin antigen-encoding RNA in this platform was as low as 50 ng to induce neutralizing antibodies.

Clinical applications of SAM (delivered by VRPs and LNPs) in the prevention of infectious disease are promising, which have been extensively reviewed elsewhere by Bloom et al. [12]. However, the applications of SAM in cancer vaccine are mainly limited to preclinical studies, with only two clinical trials (NCT00529984 and NCT01890213, Table 2) ongoing using VRP delivered antigens against colorectal cancers. The clinical and immunological benefits of SAM are still debating [7]. One major consideration that restricts SAM applicationsis the intrinsic PAMP natures, which makes it difficult to modulate the inflammatory profile, potentially limiting repeated dosing anti-tumor therapies [11].

## Delivery of mRNA Cancer vaccine

Various viral, non-viral, and cell-based vehicles have been developed to increase the delivery efficiency of mRNA cancer vaccines. Viral and cell-based vehicles have been extensively reviewed elsewhere and are not discussed in detail here [3, 7, 10, 11, 75–77]. In this section, we will focus on applications of non-viral vectors for mRNA vaccine delivery.

### Ionizable lipid nanoparticles-based mRNA delivery system

#### Rationale for lipid nanoparticles to maximize deliver efficiency and immunogenicity

**Fig. 1:**
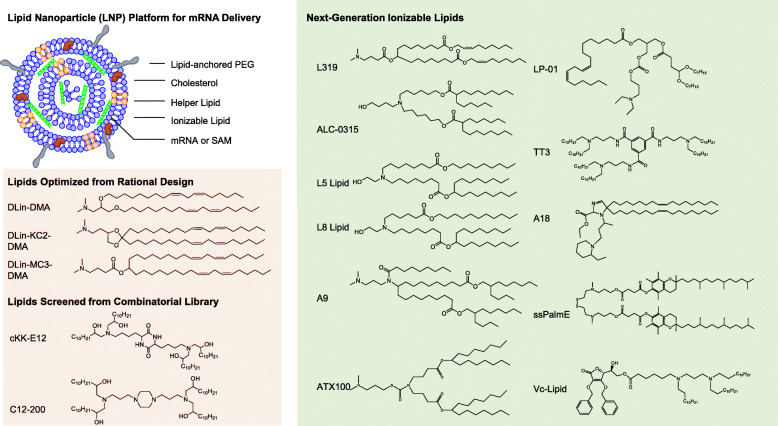
Representative LNP structure and ionizable lipids used in preclinical research and clinical trials

LNPs, which were originally designed to deliver siRNAs, have been recently applied for the delivery of mRNA and present as the most clinical-translatable non-viral delivery vehicles. LNPs are mainly composed of an ionizable amino-lipid-like molecule, a helper phospholipid, cholesterol, and lipid-anchored polyethylene glycol (PEG). The ionizable lipid is an amphipathic structure with a hydrophilic headgroup containing one or multiple ionizable amines, hydrocarbon chains capable of promoting self-assembly, and a linker that connects the headgroups with hydrocarbon chains. The ionizable lipid is designed to acquire positive charges by protonation of the free amines at low pH for two main purposes: (1) during the preparation of LNPs, the positively charged lipids can facilitate encapsulation of the negatively charged mRNA via electrostatic interaction; (2) in the acidic endosomal microenvironment upon intracellular delivery of LNPs, the positively charged lipid could interact with the ionic endosomal membrane, facilitating membrane fusion and destabilization, leading to release of mRNA from both LNPs and endosome. At the physiological pH, the ionizable lipid remains neutral, improving stability and decreasing systemic toxicity. Representative ionizable lipids include: Dlin-DMA, DLin-KC2-DMA [78], and DLin-MC3-DMA [79], which were synthesized based on rational design; C12–200 [80], and cKK-E12 [81], which were screened by high throughput screenings of combinatorial libraries; next-generation ionizable lipids, including DLin-MC3-DMA derivative L319 (Alnylam and AlCana Technologies) [82], C12–200 and cKK-E12 derivatives (Anderson’s group) [83–85], COVID-19 vaccine lipid ALC-0315 and SM-102 [86], TT3 and biodegradable derivative FTT5 (Dong’s group) [87, 88], vitamin derived lipid ssPalmE [89] and VcLNP [90], A9 (Acuitas) [91], L5 (Moderna) [92], A18 Lipid [25], ATX Lipid (LUNAR® composition, Arcturus) [93] and LP01 (Intellia Therapeutics) [94], which were mostly biodegradable (Fig. 1). Besides ionizable lipid(s), phospholipid (i.e. 1,2-dioleoyl-sn-glycero-3-phosphoethanolamine (DOPE), 1,2-distearoyl-sn-glycero-3-phosphocholine (DSPC)) and cholesterol are incorporated to improve lipid bilayer stability, aid membrane fusion and endosomal escape. The lipid-anchored PEG is incorporated to decrease macrophage-mediated clearance. More importantly, lipid-anchored PEG helps prevent particle aggregation and improve storage stability [95].

For cancer vaccine delivery, LNPs should be designed to protect mRNA from extracellular RNase degradation, and to deliver mRNA encoding antigens specifically to APCs, so to facilitate efficient antigen presentation, whilst not comprise mRNA translation. In addition, the lipid excipients used to deliver mRNA should be metabolizable and cleared rapidly, thus decreasing the potential systemic toxicity elicited from the vehicles and to allow for repeatable dosing. Ionizable lipids play crucial roles in fulfilling all these purposes. Current optimization of ionizable lipids have been focused on modulating the head group, linker and alkyl chains to adjust the acid dissociation constant (pKa), fusogenic properties, and metabolic behaviors.

Acid dissociation constant (pKa) of the ionizable amino group is strongly correlated with in vivo efficacy and immunogenicity of mRNA. The optimal pKa range for i.v. delivery of siRNAs and mRNAs are between 6.2–6.5 as screened and confirmed by Jayaraman and Sabnis et al. [79, 92]. Whereas Hassett et al. recently reported that the recommended range of lipid pKa was 6.6–6.9 for intramuscular (i.m.) injection of mRNA to induce optimal immunogenicity [54]. To achieve the targeted pKa, the head group of the ionizable lipid usually contains at least one tertiary amine or two amino groups apart [25, 79, 80]. Examples include ethanolamine headgroup in L5 lipid (pKa 6.56), dimethylamine headgroup in DLin-MC3-DMA (pKa 6.44), and 2-ethylpiperidin headgroup in A18 (pH 6.6) [25, 79, 92]. Although the weakly acidic headgroup of the ionizable lipids is an important feature for the success of the LNP, it may also contribute to the instability of the nanoparticles. According to the package insert, both Pfizer/BioNTech and Moderna COVID-19 vaccines must be stored at ultralow temperature and should be discarded after less than a day at room temperature. One hypothesis for the instability nature of LNPs is that the ionizable lipids are neutral and oil-like at storage pH (usually neutral), and thus they may not tend to stay at the interface at ambient temperature.

Besides lipid pKa, the molecular shape of the lipid may also impact mRNA expression efficiency. The hypothesis commonly acknowledged in the field is that the ionizable lipid should adopt a “cone” shape once protonated in acidic environments to facilitate endosomal escape [78]. In principle, the “cone shape” ionizable lipid, which contains lipid tails with larger cross-sectional areas than the lipid headgroups, could pair with the anionic endosomal membranes (i.e. phosphatidylserine) to form non-bilayer hexagonal H_II_ phases, resulting in fusion and disintegration of the endosomal membrane [78]. Multiple structure-activity evaluations from the high throughput lipid libraries demonstrate that incorporation of double bonds in hydrocarbon alky chains (especially cis-alkenyl group, e.g. linoleyl chains in Dlin-MC3 (KC2)-DMA) can alter the orientation of the alkyl chains, thereby enhancing the potentials to generate non-bilayer structure [96]. Linoleic acid-derived tails have been widely applied to build various ionizable or cationic lipids. For instance, Fenton and coworkers have introduced linoleic chains to the cKK-E12 based polyamine core via a ring opening reaction. The linoleic acid derivative OF-2 showed more than twice higher level of erythropoietin (EPO) expression than the cKK-E12 counterpart when i.v. injecting the EPO mRNA containing LNPs [85]. Increasing the degree of unsaturation (including alkynyl group) in the lipid tails can further enhance the fusogenicity of the lipid, and improve endosomal escape. However, stability of LNPs may be compromised [57]. Replacing alkene group with ester bond can also maintain the lipid “core shape” and the fusogenicity [92]. Finally, the alkyl chain length may also be correlated with fusogenicity. Anderson and colleagues evaluated lipids with alkyl chain length varying from C8 to C18, and showed that lipids with 12–14 carbon atoms in the tail were optimal for delivery [80]. Structural changes in the headgroup-linker region also affect the ionization behavior of the headgroup and the orientation of the alkyl chains [78]. Linker with rigidity seems to maintain a better translation efficacy (ring [25], unsaturation bond [94] and branched structures [79]).

All the above discussions focus on improving the potency of the delivery vehicles. However, safety is another index needs to be considered for chronic indications like cancer. Unfortunately, improvements in delivery vehicle potency do not always result in an enlargement of the therapeutic outcome because of the reductions in tolerated dose levels [83]. Although the U.S. FDA approved DLin-MC3-DMA lipid is well tolerated in several clinical studies, repeat dosing some of the ionizable lipid containing LNPs have shown elevated cytokine levels and increased immunogenicity [57]. A persistent theme in the development of delivery vehicles is to incorporate biodegradable design features as means to improve biocompatibility and decrease systemic off-target toxicity [82]. Ester linkages are widely used for enhancing the biodegradability of biomaterials, as it can be hydrolyzed enzymatically by esterase or lipase in tissues and intracellular compartments. Cleavage of an ester linkage within the hydrophobic chain will generate more hydrophilic by-products, carboxylic acid and alcohol that can be readily eliminated, or further metabolized by natural mechanisms [82]. In the same time, the sp^2^-carbon of the ester group helps the lipid maintain the “cone shape” to destabilize the endosomal membrane [82]. Moreover, the carboxylic acid containing derivative after hydrolysis are likely to reverse the positive charge in the amino head group, and facilitate the release of mRNA from the vehicle. For instance, L319 (DLin-MC3-DMA derivative), LP-01 and lipid 5 are reported to be cleared from the liver rapidly (half-life< 6 h) as compared to DLin-MC3-DMA (half-life > 50 h) [75, 92, 94]. However, primary ester linkages added to the lipid tail are also vulnerable to the esterase/lipase in the systemic circulation, with the potential of cleavage before delivering mRNA intracellularly, thus leading to compromised potency [92]. A balance between delivery efficiency and pharmacokinetics are a complex correlation between number/type/location of the ester bond(s) in the hydrocarbon tails, the type and structure of the headgroup and linker. Subtle change could tip the balance to one end. For instance, a combination of secondary and primary esters in the ethanolamine featured L5 lipid can maintain a satisfactory balance between expression potency and clearance. Replacing the alcohol functionality with dimethylamine in the head group or moving the primary ester closer to the nitrogen group all introduce loss of delivery efficiency [92]. In some cases, introducing of ester bond can modulate the expression of protein in different cell types. For example, OF-Deg-Lin induced protein expression selectively in the B cells of the spleen [84]. Therefore, rational design of biodegradable lipids could offer better control over clearance rate and expression selectivity [75].

In addition to chemical modifications of the ionizable lipids, formulation of LNPs were also optimized to potentiate antigen expression and adaptive immune response. Kauffman et al. have used design of experiment (DOE) to investigate the impact of ionizable lipid ratios, the type of helper lipids on the mRNA delivery efficiency [97]. The researchers found out that incorporation of DOPE as the helper lipid into cKK-E12 LNP could improve mRNA but not siRNA expression. The same group later evaluated the impact of lipid length, PEG molecular weight and mole percentage of lipid-anchored PEG in LNPs on the distribution patterns of the encapsulated siRNA in vivo. The highest liver distribution was observed when 0.75% of C18-PEG1000 were incorporated into C12–200 LNP formulations [98]. Miao et al. have evaluated the mRNA expression using LNP containing combinations of different ionizable lipids, and indicated that combining a protein binding ionizable lipids with a lipid of high fusogenicity could potentiate mRNA expression [18]. Organ specificity can also be tuned by modifying the lipid formulations. For instance, Kranz et al. figured out that decreasing the ratio of cationic lipid to DOPE in the mRNA loaded lipoplex could shift mRNA expression from the lungs towards spleen. Based on this rationale, they have developed lipoplexes that systemic delivered mRNA vaccine to splenic dendritic cells [18].

#### Mechanistic studies and additional functional modifications of LNPs

The rationales and mechanisms behind LNP internalization, endosomal escape and organ/cell-selective delivery have been widely investigated by multiple groups using either siRNA or mRNA as the delivered molecules [81, 92, 99–102]. In brief, apolipoprotein E(ApoE) or albumin-based receptor mediated endocytosis and non-specific micropinocytosis are two major mechanisms responsible for the update of mRNA/siRNA loaded LNPs [57, 100, 103]. To improve the specific delivery of LNPs to APCs, targeting ligand was further added to modify the LNPs. For instance, mannose-cholesterol conjugates (MPn-CHs) was added to LNPs post formulation preparation through click reaction with the PEG units [104]. The mannose modified LNPs were shown to impove the uptake of the particles in DCs through mannose receptor CD206. Insufficient release of mRNA/siRNA from endosomal compartment has been considered as the predominant obstacle that limits the expression of mRNA/siRNA delivered by LNPs. Intracellular trafficking of LNP loaded siRNA/mRNA have been visualized using electron microscope (EM) [100], high-dynamic range live-cell imaging confocal [102], single-molecule fluorescence in situ hybridization (FISH) [92], etc.. By directly detecting colloidal-gold particles conjugated to siRNAs using EM, Gilleron and coworkers demonstrated that only 1–2% of siRNA delivered by DLin-MC3-DMA LNPs could escape from the endosomes into cytosols. Moreover, the cytosolic release of siRNA/mRNA only occurs during a narrow window of time when the LNPs reside in early matured endosomes, as reported by both Wittrup et al. and Gilleron et al. [100, 102]. Ionizable lipids or helper lipids with increased fusogenicity have been incorporated into LNPs to improve the endosomal escape of mRNA/siRNAs. For instance, Moderna L5 LNPs showed 6-fold higher rate of endosomal escape as compared to the DLlin-MC3-DMA LNPs [92].

Immunogenicity of the delivery materials were also evaluated and applied to boost immune response of the cancer vaccines. Miao et al. have developed a group of ionizable lipids containing cyclic amino head groups, isocyanide linker, and linoleic acid derived alkyl tails that provides adjuvant activities independent of the encapsulated mRNA [25]. The cyclic amino head and isocyanide linker of the lipids directly bound to STING (stimulator of interferon genes) protein and triggered the activation of Type I IFNs, leading to activation of humoral and cellular immune response.

#### LNP mRNA vaccine from formulation to manufacturing

The conventional benchtop formulation process for LNPs includes direct mixing, thin film, ethanol injection, which are usually labor intensive, lack of scalability and reproducibility. More recently, great control was achieved over the mixing process when performed by T-junction mixing, microfluidic using microfluidic hydrodynamic focusing (MHF) or Staggered herringbone mixing (SHM). The rationales and advantages of each rapid mixing methods were summarized by Pieter R. Cullis and coworker elsewhere [105]. In brief, these chip-based microfluidic devices mix two laminar flows, the RNA-containing aqueous phase and the lipids-containing ethanol phase, through a confined microchannel equipped with chaotic mixers at a controlled speed, leading to rapid diffusion and self-assembly of mRNA-LNP at the interface [106]. High encapsulation efficiency (> 90%) and low polydispersity can be achieved by rapid laminar flow mixing. The laminar flow rapid mixing method is scalable for GMP production of LNPs. For instance, Precision NanoSystems team produced GMP microfluidic product of LNPs using the NanoAssemblr GMP system and a TrM (NxGen500) cartridge [74]. With the recent approval of two mRNA vaccines for prevention of COVID-19 from Pfizer/BioNTech and Moderna, rapid GMP manufacturing of COVID-19 vaccine (including mRNA and LNP manufacturing) are highly required. For instance, BioNtech/Pfizer were committed to produce vaccines at 6 manufacturing sites to achieve 570 million doses for support dosing in 13 countries. This further supports the feasibility of rapid production of mRNA vaccines to fulfill commercial requirement.

### Polymer-based mRNA delivery system

Polyamines, dendrimers, biodegradable copolymers are commonly used polymer-based materials for mRNA delivery. Polymer-based delivery systems tend to have lower purity due to high polydispersity, lower clearance rate due to large molecular weight, and worsen toxicity profile due to condensed charge density compared to synthetic LNPs, and they are not as clinically advanced for mRNA delivery as ionizable lipids [75, 77]. To improve the tolerability and stability of the polymeric platforms, structural modifications, which include incorporating of lipid tails, hyperbranched groups and biodegradable moieties have been evaluated [77, 107–109].

Polyethylenimine (PEI) is one type of cationic polymer commonly used for nucleic acid delivery. The commercial linear PEI derivative, jetPEI®, has already been used for mRNA in vivo/in vitro transfection. A PEI formulation of SAM encoding the hemagglutinin antigens from influenza virus strains stimulated high antibody titer after i.m. vaccination in mice [68]. However, PEI is known with the severe systemic toxicity and low biodegradability due to the high charge density and molecular weight. Low-molecular-weight PEI modified with fatty chains has been used for siRNA/mRNA delivery to reduce toxicity [110, 111]. Polysaccharide and derivatives are another group of commonly used cationic polymers. McCullough and coworkers have condensed SAM-encoding influenza virus hemagglutinin and nucleoprotein with chitosan, a commonly used polysaccharide excipient. The researchers observed expression of antigens in DCs after s.c. injection of the particles [112]. Son et al. reported the use of polysaccharides derived from the microbial cell wall to form a flexible core-shell structure to encapsulate mRNA and promote DC activation in vivo [10].

Polyamidoamine (PAMAM) or polypropylenimine based dendrimer is another group of cationic polymer material used for mRNA delivery. Khan et al. developed fatty chain modified PAMAM dendrimers for delivery of siRNA systemically to lung endothelial. The same group later used the same delivery vehicle and delivered antigen-encoding SAMs. The researchers showed that the single dose, adjuvant free i.m. delivered SAM protected mice from lethal challenge of Ebola, H1N1 influenza, Toxoplasma gondii, respectively [113]. Islam and coworkers utilized a modified PAMAM dendrimers, PLGA and ceramide PEG to formulate polymer-lipid hybrid nanoparticles to deliver phosphate and tensin homolog mRNA in vivo [114]. In a later study, they utilized the same vehicle to deliver OVA mRNA vaccine together with a fatty acid modified TLR7/8 agonist C16-R848, and showed the combination formulation could boost a strong antitumor immunogenicity [55].

Biodegradable polymers were developed to increase the clearance while decrease the charge induced toxicity of the delivery vehicles. Poly (beta-amino) esters (PBAEs) are biodegradable polymers used for siRNA/mRNA delivery. Kaczmarek et al. co-formulated PBAEs with PEG-lipid to improve serum stability and increase mRNA delivery efficiency. Besides adding lipid to the PBAE formulations, hyperbranched PBAEs were utilized to stabilized the formulation and to deliver mRNA to lung endothelium via i.v. injection [107, 115], and to lung epithelium vial inhalation [108]. Other biodegradable polymers have been designed to achieve lower toxicity and selective delivery of mRNA to different organs. Kowalski et al. demonstrated that biodegradable amino polyesters (APEs), synthesized using ring-opening polymerization of various lactones, were capable of tissue-selective mRNA delivery [109]. Moreover, bio-reducible poly (CBA-co-4-amino-1-butanol) (pABOL), developed by Blakeny et al., were used to deliver haemagglutinin-(HA-) encoding SAM in mice [69].

Charge altering polymers have also been explored for mRNA vaccine delivery. Wender’s group developed a library of charge-altering releasable transports (CARTs) that utilized poly(carbonate)-β-(α-amino ester)s. CARTs undergo dynamic change from an ester to amide rearrangement. As a result, the cationic poly α amino ester backbone is gradually changed into neutral small molecules (diketopiperazine), providing a mechanism for release of mRNA, and avoiding tolerability issues associated with persistent polycations. The CART polymers facilitated mRNA transfection into lymphocytes including T cells [116–118].

### Peptide-based mRNA delivery system

The cationic peptide, protamine has been used in many early studies for the delivery of mRNA vaccines. Protamine spontaneously condenses mRNA through electrostatic interaction, protecting the encapsulated mRNA from degradation by extracellular RNases. The protamine-mRNA complexes can also function as adjuvant, activating TLR7/8 to elicit Th-1 type immune response [119]. However, protamine-mRNA complexes alone showed suboptimal translation efficiency, which might be due to an excessively tight interaction between protamine and mRNA. This concern has been solved by a two-compartment formulation, RNActive®, developed by CureVac AG. The researchers combined protamine-mRNA complexes (50%) with naked antigen-coding mRNA(s) (50%). The protamine complexes act only as adjuvant, while the nucleoside modified mRNA acts as antigen producer. RNActive® encapsulating TAAs-encoding mRNAs are currently being evaluated in several phase I/II clinical trials treating multiple solid tumors [5, 120–122]. Most RNActive® vaccines are well tolerated and immunogenic in patients, some of them have shown moderate antitumor efficacy.

Cationic cell-penetrating peptides (CPPs) can complex with RNA. Although their cell-uptake mechanisms are not fully understood, it is hypothesized that CPPs may facilitate clustering of the negative charged glycosaminoglycans on the cell surface, and trigger micropinocytosis [75]. RALA peptide is an amphipathic arginine-rich CPP with positively charged arginine residues on one end and neutral leucine residues on the other [123, 124]. Researchers indicated that the peptide condensed mRNA complexes enabled mRNA delivery and expression in DCs, subsequently eliciting potent cytolytic T cell responses after i.d. injection of the ex-vivo loaded DCs [124]. Furthermore, D-amino acid-based truncated protamine was fused with a short CPP called Xentry. This fusion peptide with combined positive and cell penetrating features was used to deliver a cystic-fibrosis transmembrane regulator (CFTR) mRNA into several human epithelial cells in vitro [125]. In another study, Zhang et al. used cholesterol-modified cationic peptide DP7 with transmembrane structure and immunoadjuvant function to modify the DOTAP liposomes. This DOTAP/DP7-C liposomes efficiently transferred mRNA into different type of DCs in vitro. Subcutaneous injection of neoantigen-encoding mRNA loaded in DOTAP/D7-C liposomes significantly inhibited the growth of LL2 [126]. Similarly, an alpha-helical cationic CPP “KALA” was combined with the vitamin E-scaffold (ssPalmE)-LNP to achieve higher protein expression and increased proinflammatory cytokines secretion in DCs, functioning as a potent ex vivo DCs-based RNA vaccine platform [127]. Besides positive charged CPP, negative charged GALA peptide has been used as a targeting ligand, that click onto LNPs/polyplexes to improve the cell penetration of mRNAs [128].

### Other formulations used in mRNA delivery

In additional to ionizable lipid composed LNP system, cationic lipid composed liposomes, lipoplexes and cationic emulsions (CNE) are the very first generation of carriers used for mRNA delivery both preclinically and in clinical trials. DOTMA (1,2-di-O-octadecenyl-3-trimethylammonium propane) and DOTAP (1,2-dioleoyl-3-trimethylammonium-propane) are two most widely used cationic lipids [77]. These lipids remain positively charged at all physiological pH, and can easily condense anionic mRNA. A combination of DOTMA/DOTAP with fusogenic helper lipid DOPE to form lipoplexes have been used by BioNTech in their Lipo-MERIT cancer vaccine platform. The ratio of cationic lipid and DOPE can be tuned to selectively target splenic APCs for mRNA vaccine delivery [18]. Promising therapeutic outcome has been seen in several ongoing clinical trials treating metastatic melanoma (summarized in later section). In addition, DOTAP containing cationic CNE, which is derived from the Novartis’s first FDA approval CNE MF-59 have been used for mRNA delivery. For instance, cationic CNE was used by Brito et al. to encapsulate SAM [129]. The CNE was prepared by mixing an aqueous phase containing buffer and Tween 80 with an oil phase containing Sorbian tioleate (Span 80), DOTAP, and oil squalene. The researchers have shown that the protein expression of mRNA delivered by the CNE through i.m. administration was similar to a viral vector. The mRNA CNE vaccine was well tolerated and immunogenic in a variety of models. DOTAP containing liposomes were also used as a shell for encapsulating mRNA in core-shell structures. For instance, Huang lab has developed lipid/calcium/phosphate (LCP) system using calcium phosphate as the core to condense mRNA, and PEGylated DOTAP/DOPE liposome as the shell [130]. The researchers delivered MUC-1 (TAA of the triple negative breast cancer) mRNA to 4 T1 breast cancer bearing mice, and observed potent antigen-specific T cell activation and improved antitumor efficacy. Moreover, Lipid-Polymer-RNA lipopolyplexes (LPR), functionalized with a tri-antenna of α-d-mannopyranoside (triMN-LPR) can specifically bind to human and mouse DC, provide high induction of a local inflammatory response after i.d. injection [131]. Another LPR system consisting of poly (β-amino ester) polymer/mRNA core encapsulated into a 1,2-dioleoyl-sn-glycero-3-ethylphosphocholine/1,2-dioleoyl-sn-glycero-3-phosphatidyl-ethanolamine/1,2-distearoyl-sn-glycero-3-phosphoethanolamine-N-[amino(polyethyleneglycol)-2000] (DOPC/DOPE/DSPE-PEG) lipid shell were developed by Persano et al. to deliver mRNA into DC through micropinocytosis. Results shown that the LPR induced potent antigen response [132]. A similar LPR platform is currently being evaluated in phase I clinical trial carrying mRNA encoding neoantigens to treat metastatic melanoma by Stemirna Therapeutics.

In additional to non-viral deliver system, naked mRNA has been directly injected i.d. or intranodally as anti-cancer vaccine or ex vivo loaded into DCs for cancer vaccinations. The naked mRNA vaccines and DC-based mRNA vaccines have been widely evaluated in clinical trials with some optimistic therapeutic outcome for cancer treatment. However, the strategies are either suffered from insufficient antigen expression, complicated in vitro processing or batch to batch variabilities [11]. Clinical overview, advantages and limitations of these two types of vaccines were discussed elsewhere [3, 7, 77], therefore will not be covered in detail in the current review.

### Injection routes mRNA Cancer vaccines

Local injections, including i.m., s.c., i.d., are the commonly used injection routes for mRNA cancer vaccines. Representative examples include: i.m. injection of PAMAM loaded OVA mRNA for melanoma treatment in mice [55], Moderna LNPs optimized for i.m injection of mRNA vaccines [54], s.c. injection of peptide modified DOTAP liposomes, s.c. injection of LNPs with optimized lipid compositions and lipid structures for antitumor vaccinations [26], i.d. injection of LPR to boost anti-cancer immunity in multiple mouse models [131].

Intramuscular administration is often preferred due to the flexibility of injection volume, the ease of dosing and the lack of safety concern, with limited risk for adverse reactions at the site of injection [133]. However, vaccine delivered to the skin as a highly immunocompetent site has long been considered a strategy to augment vaccine response [133]. Ols and coworkers have investigated the impact of vaccination route (mainly i.m. and s.c.) on antigen trafficking and immune response in Rhesus Macaques using fluorescently labeled HIV-1 envelope glycoprotein trimers displayed on liposomes. The researchers found that both s.c. and i.m. routes induced efficient immune cell infiltration, activation and antigen uptakes. Though the immunogenicity is tightly restricted to the injection site, and antigen also transported to different lymph nodes depending on route, these early differences failed to convert into significant differences in the magnitude or quality of antigen-specific immune response. Despite this, the expression level and inherent innate immunity of mRNA might be influenced by the routes of administration, subsequently leading to different intensity of immune response. Using the most translatable carrier LNPs as an example, Pardi et al. have evaluated the expression kinetics of nucleoside modified mRNA in mice through various routes of administration [134]. Their findings demonstrated that i.m. and i.d. delivery of mRNA LNPs resulted in the longest duration of mRNA translation (half-life > 20 h) followed by s.c. (half-life ~ 15 h) and i.v. (half-life ~ 7 h). Whereas, s.c. and i.m showed higher protein expression level as compared to i.d [134]. The differences in magnitude and length of protein expression from different routes of administration may directly impact the intensity of immunogenicity, which required detailed evaluations in the future studies. As covered in Section 3, the kinetics between TCR activation and IFN signaling can also be dependent on the route of mRNA administration, ultimately impacting the potency of T cell activation. Based on this perspective, systemic mRNA vaccination through i.v. injection is more likely to promote a favorable CD8^+^ T cell response and circumvent the detrimental impact of mRNA inherent innate immunity. As a result, vaccination through i.v. injection has been used by several researchers and companies [18, 22]. However, one needs to concern about the potential systemic toxicity generated from i.v. vaccination. Until now, s.c. and i.m. injections are still the two major injection routes for mRNA cancer vaccination in clinical trials, due to their less invasive nature; however, other injection routes, including intranasal, and intranodal have been widely studied for mRNA vaccine delivery [135].

## Clinical overview of mRNA Cancer vaccines

Transfection of mRNA into DCs for adoptive transfer was the first mRNA based therapeutic cancer vaccine entering clinical trial [75]. Although DC-based mRNA vaccine therapeutics still account for majority of mRNA cancer vaccines in clinical trials, IVT mRNA-based immunotherapies delivered by non-viral vectors are extensively explored recently as a result of the promising antitumor outcomes collected from preclinical studies, with CureVac, BioNTech and Moderna as pioneers in the campaign. A group of IVT mRNA-based immunotherapies investigated in clinical trials are mRNAs encoding immunostimulants (Table 1, e.g. IL-12, IL32, OX40L, CD40L, CD70, etc.), which are injected intratumorally or intranodally to modify the suppressive tumor microenvironment. These immunostimulants are not considered as cancer vaccines, but are usually co-administered with cancer vaccines or other immunotherapeutic agents (e.g. checkpoint blockade modulators) and act as adjuvants to augment humoral and cellular response. Multiple IVT mRNA-based cancer vaccines are currently tested in clinical trials, either encoding personalized neoantigens, or a cocktail of TAAs (Tables 2 and 3). Deliver systems for these mRNA-based cancer vaccines include lipid polyplexes, CNEs, LNPs or protamine. Local injection, such as i.m., s.c. and i.d. are major administration routes for mRNA vaccines in the clinical studies, whereas the BioNTech product, Lipid-MERIT (DOTAP (or DOTMA)/DOPE lipoplex as deliver system) is vaccinated intravenously. As discussed earlier, the ratio between DOTAP and DOPE can be optimized to allow specific delivery of mRNA to splenic APCs, and induce potent antigen-specific response. mRNA vaccines have been applied to treat aggressive, less accessible and metastatic solid tumors, including non-small cell lung cancers (NSCLC), colorectal carcinoma (CRC), melanoma, etc. For early proof of concept studies, mRNA vaccine has also been tested in treating glioblastoma. In most clinical trials, mRNA cancer vaccines are further combined with checkpoint modulators or cytokine cocktails to augment antitumor efficacy.

**Table 1:** Clinical Trials of mRNA Encoding Immunostimulants

NCT Number	Status	Phases	Disease	mRNA and Interventions	Formulation Type	Route	Combo	Sponsor (s)	Study Results
NCT03788083	Recruiting	Phase 1	Early-stage Breast Cancer	Trimix mRNA (mRNA encoding CD40L, CD70, acTLR4)	Synthetic naked mRNA	Intratumoral	NA	Universitair Ziekenhuis Brusse, eTheRNA immunotherapies	Not available
NCT03394937	Recruiting	Phase 1	Melanoma (resected)	ECI-006: a. Trimix mRNA, b. mRNA encoding TAAs: tyrosinase, gp100, MAGE-A3, MAGE-C2, PRAME	Synthetic naked mRNA	Intranodal	Trimix mRNA + TAA	eTheRNA immunotherapies	ECI-006 is well tolerated. Vaccine-induced immune responses were detected in 4/10 and 3/9 patients treated with low (600 μg) and high dose (1800 μg). ECI-006 shown immunogenic in a portion of patients.
NCT01066390	*Completed*	Phase 1	Stage III/IV Malignant Melanoma (Previously treated, unresectable)	TriMixDC-MEL a. DC electroporated with TriMix mRNAs, b. TAAs: MAGE-A3, MAGE-C2, tyrosinase, gp100	DC-based	Autologous DC treatment (i.v. and i.d.)	Trimix mRNA + TAA	Universitair Ziekenhuis Brussel	Immunotherapy with TriMixDC-MEL is safe and immunogenic. Antitumor activity with durable disease control is observed. Antigen-specific CD8^+^T-cells were detected in the blood of 4 of 5 patients.
NCT01676779	*Completed*	Phase 2	Melanoma (disease free following macrometastases)	TriMixDC-MEL a. DC electroporated with TriMix mRNAs b. TAAs: MAGE-A3, MAGE-C2, tyrosinase, gp100	DC-based	Autologous DC treatment (i.v. and i.d.)	Trimix mRNA + TAA	Universitair Ziekenhuis Brussel	TriMixDC-MEL is tolerable (symptom: transient local skin reactions, flu-like symptom, post-infusion chills), and may improve the 1-year disease-free survival rate (71% disease free in treatment group vs 35% in control arm).
NCT01302496	*Completed*	Phase 2	Stage III/IV Malignant Melanoma (Previously treated, unresectable)	TriMixDC-MEL and i.v. CTLA-4 inhibitor ipilimumab	DC-based	Autologous dc therapeutics (i.v. and i.d.)	Trimix mRNA + TAA + Checkpoint inhibitor	Bart Neyns|Vrije Universiteit Brussel|Universitair Ziekenhuis Brussel	T-cell stimulation were shown in 12/15 patients. Immune responses were stronger in patients with complete or partial response. Multifunctional CD8+ T-cell responses were detected either elicited by TriMixDC-MEL IPI or on subsequent pembrolizumab treatment, may provide a benchmark for the level of immune stimulation needed to achieve a durable clinical remission.
NCT03323398	Recruiting	Phase 1/2	Relapsed/ Refractory Solid Tumor Malignancies or Lymphoma	mRNA-2416 (mRNA encoding OX40L), alone (Phase I) or in combination with i.v. PD-L1 inhibitor, Durvalumab (Phase 2)	LNP	Intratumoral	mRNA LNP + Checkpoint inhibitor	ModernaTX, Inc.	Intratumoral mRNA-2416 is tolerable at all dose levels when dosed alone. Analyses of tumor post-treatment demonstrate increased OX40L protein expression, elevated PD-L1 levels and pro-inflammatory activity.
NCT03739931	Recruiting	Phase 1	Dose Escalation: Relapsed/Refractory Solid Tumor Malignancies or LymphomaDose Expansion: Other solid tumors	mRNA-2752 (mRNA encoding OX40L, IL-23, IL-36Ƴ), alone (Phase I) or in combination with i.v. PD-L1 inhibitor, Durvalumab (Durva, Phase II)	LNP	Intratumoral	mRNA LNP + Checkpoint inhibitor	ModernaTX, Inc., AstraZeneca	Intratumoral mRNA-2752 given as monotherapy and in combination with PD-L1 inhibitor is tolerable at all dose levels studied, and administration can be associated with tumor shrinkage (52% Tumor reduction, 0.5 mg mRNA-2752 with durva in bladder carcinoma). Elevated IFN-γ, TNF-α, and PD-L1 levels were detected.

**Table 2:** Clinical Trials of mRNA Encoding TAAs

Brand	NCT Number	Status	Phases	Disease	Antigen	mRNA	Formulation Type	Route	Combo	Sponsor (s)	Study Results
CV9201	NCT00923312	*Completed*	Phase 1/2	Stage IIIB/IV NSCLC	MAGE-C1, MAGE-C2, NY-SEO-1, survivin, 5 T4	mRNA	RNActive, (Protamine)	i.d.	NA	CureVac	CV9201 was well-tolerated and immune responses were detected after treatment. Median progression-free and overall survival were 5 and 10.8 months
CV9202	NCT03164772	Recruiting	Phase 1/2	NSCLC	NY-ESO-1, MAGE-C1, MAGE-C2, 5 T4, survivin, MUC1	mRNA	RNActive, Protamine	i.d.	Durvalumab; Tremelimumab	CureVac	CV9202 was well-tolerated, and antigen specific immune responses were detected in majority of patients (84%)
CV9103	NCT00831467	*Completed*	Phase I/2	Prostate cancer	PSA, PSCA, PSMA, STEAP1	mRNA	RNActive, Protamine	i.d.	NA	CureVac	CV9103 is well tolerated and immunogenic
CV9104	NCT01817738	*Terminated*	Phase I/2	Prostate cancer	PSA, PSCA, PSMA, STEAP1, PAP, MUC1	mRNA	RNActive, Protamine	i.d.	NA	CureVac	Terminated due to insufficient activities
BNT111 (Lipo-MERIT)	NCT02410733	Active, not yet recruiting	Phase 1	advanced melanoma	NY-ESO-1, MAGE-C3, tyrosinase, gp100	mRNA	Lipo-MERIT, DOTMA (DOTAP)/ DOPE lipoplex	i.v.	NA	BioNTech	Not available
IVAC	NCT02316457	Active, not yet recruiting	Phase 1	TNBC	3 TAAs selected	mRNA	Lipo-MERTI, DOTMA(DOTAP)/DOPE lipoplex	i.v.	NA	BioNTech	Not available
Not available	NCT01995708	Active, not yet recruiting	Phase 1	malignant melanoma	CT7, MAGE-A3, and WT1 mRNA-electroporated Langerhans cells (LCs)	dendritic cell (DC)-loaded mRNA	CT7, MAGE-A3, and WT1 mRNA-electroporated Langerhans cells (LCs)	i.d.	NA	Memorial Sloan Kettering Cancer Center	Not available
Not available	NCT00204516	*Completed*	Phase 1/2	melanoma	TAA for melanoma (Melan-A, Mage-A1, Mage-A3, survivin, GP100, and tyrosinase)	naked mRNA	naked mRNA	i.d.	GM-CSF	The Norwegian Radium Hospital	Not available
NA	NCT01278940	*Completed*	Phase 1/2	melanoma	TAA-transfected DC	dendritic cell (DC)-loaded mRNA	DC loaded mRNA	i.d. or i.n.	IL-2	Oslo University Hospital	Not available
AVX701	NCT01890213	*Completed*	Phase 1	Stage III CRC	an alphavirus replicon (VRP) encoding the protein (CEA)	SAM	VRP	i.m.	NA	AlphaVax	Not available
AVX701	NCT00529984	*Completed*	Phase 1/2	Advanced or metastatic CEA expressing solid tumor	an alphavirus replicon (VRP) encoding the protein (CEA)	SAM	VRP	i.m.	NA	AlphaVax	Five-year survival for patients with stage IV and stage III cancer was 17, 75%, respectively. All patients shown CEA-specific humoral immunity. CEA-specific, IFNγ-producing CD8 + granzyme B + TCM cells were increased. So, VRP-CEA induces antigen-specific effector T cells while decreasing Tregs, suggesting favorable immune modulation.

**Table 3:** Clinical Trials of mRNA Vaccines Encoding Neoantigens (Neo-Ag)

Brand	NCT Number	Status	Phases	Disease	Antigen /mRNA	Formulation Type	Route	Combinations	Sponsor/Collaborators	Study Results
IVAC MUTANOME, RBL001/RBL002	NCT02035956	*Completed*	Phase 1	Advanced Melanoma	Neo-Ag/TAA (mRNA)	naked mRNA	ultrasound- guided i.n.	NA	BioNTech	60% of the 125 selected neoepitopes elicited a T- cell response. The vaccination was very well tolerated.
RO7198457	NCT03289962	Recruiting	Phase 1	Melanoma, NSCLC, Bladder Cancer, CRC, Breast Cancer etc.	Neo-Ag (mRNA)	Lipo-MERIT	i.v.	Atezolizumab (infusion)	BioNTech, Genentech	RO7198457 combined with atezolizumab was generally well tolerated; RO7198457 in combination with atezolizumab induced the release of pro-inflammatory cytokines and peripheral T-cell responses in the majority of patients
	NCT04267237	Recruiting	Phase 2	NSCLC	Neo-Ag (mRNA)	Lipo-MERIT	i.v.	Atezolizumab	Hoffmann-La Roche	NA
	NCT03815058	Recruiting	Phase 2	Advanced Melanoma	Neo-Ag (mRNA)	Lipo-MERIT	i.v.	Pembrolizumab (infusion)	BioNTech, Genentech	NA
	NCT04486378	Recruiting	Phase 2	Stage II and III CRC (surgically resected)	Neo-Ag (mRNA)	Lipo-MERIT	i.v.	NA	BioNTech	NA
	NCT04161755	Recruiting	Phase 1	Pancreatic Cancer (surgically resected)	Neo-Ag (mRNA)	Lipo-MERIT	i.v.	Atezolizumab, FOLIFIRINOX	Memorial Sloan Kettering Cancer Center, Genentech	NA
mRNA-4157	NCT03313778	Recruiting	Phase 1	Mono-: resected solid tumors; Combo: unresectable solid tumor	Neo-Ag (mRNA)	LNP	i.m.	Pembrolizumab (infusion)	Moderna, Merck	mRNA-4157 is safe and well tolerated at all tested dose levels. Clinical responses were observed when dosing combined with Pembrolizumab. Neoantigen-specific T cells have been detected
	NCT03897881	Recruiting	Phase 2	Complete Resection of High-Risk Melanoma	Neo-Ag (mRNA)	LNP	i.m.	Pembrolizumab (infusion)	Moderna, Merck	Not available
mRNA-5671/Merck V941	NCT03948763	Recruiting	Phase 1	CRC, NSCLC, pancreatic cancer	KRAS mutations: G12D, G12V, G13D, G12C (mRNA)	LNP	i.m.	Pembrolizumab (infusion)	Moderna, Merck	Not available
Not available	NCT03468244	Recruiting	Phase 1	Advanced Esophageal Squamous Carcinoma; Gastric Adenocarcinoma; Pancreatic Adenocarcinoma; Colorectal Adenocarcinoma	Neo-Ag (mRNA)	lipopolyplex	s.c.	NA	Changhai Hospital, Stemirna Therapeutics	Not available

Although SAMs are an appealing alternative to mRNA-based vaccine due to their inherent self-amplifying property, clinical investigation for cancer applications is only limited to early evaluation of VRPs. With the recent advancing of cancer immunotherapies, specifically the discovery of neoantigens, development of personalized vaccines and checkpoint blockade modulators, numerous improvements have been done to demonstrate the viability of mRNA vaccines to combat cancer [11, 136]. In this section, we will discuss mRNA applications as immunostimulants and cancer vaccines, compare the delivery of mRNA encoding TAAs and neoantigens, as well as discuss the advantages of personalized vaccines and combination immunotherapies with checkpoint blockade modulators.

### mRNA encoding Immunostimulants

Immunostimulants are commonly cytokines or chemokines that induce APC maturation and activation, activate T-cell mediated immunity and adjust the dysfunctional immune tumor microenvironment (Table 1). Intra-tumoral, intranodal, i.d and i.v. routes of administration have been used dosing of mRNA encoding immunostimulants, with most evaluations are currently in Phase I/II to assess the tolerability as monotherapy or combination therapy with other moieties, including either PD-1/PD-L1 antibodies or cancer vaccines.

One pioneer player in this field is eTheRNA immunotherapies. The company has developed a TriMix mRNA-based adjuvant that consists of three naked mRNA molecules, encoding the costimulatory molecule CD70 to induce activation of CD8^+^ T cells, the activation stimulator CD40 ligand (CD40L) to activate CD4^+^ T cells, and the constitutively active TLR4 (caTLR4) to facilitate DC antigen presentation [137]. The naked TriMix mRNA and ex-vivo DC loaded TriMix mRNA evaluated in multiple clinical trials are generally well tolerated and immunogenic [58, 138, 139]. Delivery of mRNA encoding TAAs (e.g. MAGE-A3, tyrosinase, gp-100 and melano-A/MART-1) and TriMix mRNA to DCs, ex vivo or in situ, can reprograms them to mature APCs, and subsequently prime the function of T cells. In two Phase II studies for treating patients with stage III/IV melanoma, either as standalone product (TriMix mRNA plus TAA mRNA, so called TriMixDC-MEL) or combined with a CTLA-blocking monoclonal antibody ipilimumab checkpoint inhibitor, the products were able to elicit powerful immune response, in turn resulted in promising clinical response and prolonged disease-free survival rate [58, 138] (NCT01676779, NCT01302496).

Another pioneer player Moderna has developed two mRNA products encapsulated in the LNP platform for intratumoral immunostimulatory activities. These two products are currently evaluated in Phase I clinical trials to determine the safety and tolerability of repeated dosing. One product is mRNA-2416, using mRNA encoding OX40L, either dosed alone or in combination with i.v. administered PD-L1 inhibitor durvalumab for treatment of lymphoma and metastatic ovarian cancer (NCT03323398). The other product is mRNA-2752, which is composed of OX40L/IL-23/IL-36Ƴ mRNAs for treatment of lymphoma (NCT03739931). In mRNA-2752 cocktail, OX40L composes the positive secondary signals to enhance T cell effector function, expansion and survival. IL-36Ƴ functions as proinflammatory cytokines to further boost anticancer responses. IL-36 Ƴ also correlates with good prognosis in cancer patients, and induces a favorable T helper 1 type TME change. IL-23 (IL-12 family members) can act as the central coordinators and bridge innate and adaptive immunities [60]. Besides IL-23, IL-12 mRNA is also commonly used for improved antitumor immunity. Moderna is collaborating with AstraZeneca, and planning to develop MED I1191 (IL-12 mRNA) through intratumoral injection. Meanwhile, BioNtech’s cationic lipoplexes loaded BNT151 (nucleoside modified IL-12 mRNA) was also under pre-clinical evaluation for amplification of vaccine induced T cell response through i.v. administration. These two products are planned for initiation of Phase I clinical studies in 2021.

It should be noted that several small molecule drugs, especially the kinase inhibitors such as sunitinib, are potent modifiers of the suppressive tumor microenvironment. Sunitinib base formulated in a nanoemulsion, when administered i.v., significantly decreased the content of regulatory T cells (Treg) and myeloid-derived suppressor cells (MDSC) and increased T-cells in the melanoma, and enhanced the tumor growth inhibition of a therapeutic vaccine [140].

### mRNA vaccine encoding tumor associated antigens

One of the key obstacles to the development of an effective cancer vaccine is the difficulties in antigen selection. Cancer vaccines can be designed to target TAAs that are preferentially expressed in malignant cells. For instance, tyrosinase, gp100, MAGE-A3, MAGE-C2 have been identified as TAAs for melanoma. A cocktail of mRNA vaccines encoding all the TAAs have been used to treat metastatic melanoma in multiple clinical studies (Table 2).

One well-known example of mRNA vaccine platform falls into this category is Lipo-MERIT [141]. As mentioned earlier, Lipo-MERIT is fabricated by complexing mRNA with cationic lipid such as DOTMA or DOTAP. The lipoplexes with a cationic lipid: DOPE (helper lipid): mRNA ratio of 1.3:2 (≈250 nm in size and ≈30 mV in zeta potential) were shown to efficiently target the splenic DCs in mice and led to strong activation of NK, B, CD4^+^, CD8^+^ T cells, subsequently resulting in potent immunotherapeutic efficacy in multiple mouse cancer models and was translated into clinics. In one clinical study (NCT02410733), the mRNA vaccine (BNT111) encoding four TAAs (NY-ESO-1, MAGE-A3, tyrosinase, and TPTE) was evaluated in patients bearing advanced melanoma. Results demonstrated that three patients generated T cell responses against NY-ESO-1, two of which also showed responses against MAGE-A3 [18]. Recently, BioNTech announced a strategic collaboration with Regeneron to initiate the phase II clinical trial combining BNT111 with Regeneron Libtayo (cemiplimab), a fully humanized anti-PD-1 therapy in patients with anti-PD1-refractory/relapsed, unresectable Stage III or IV cutaneous melanoma [142].

Another player in the campaign is CureVac AG. CureVac have developed mRNA vaccine CV9202, containing mRNAs encoding 6 different NSCLC TAAs (MUC-1, surviving, Trophoblast Glycoprotein, NY-ESO-1, MAGE-C1 and MAGE-C2). The naked TAA mRNA vaccines were co-delivered with protamine/mRNA complexes, which are known to have self-adjuvant properties as discussed earlier. The new collaboration focused on CureVac’s CV9202 in early clinical development, in combination with afatinib for patients with advanced or metastatic epidermal growth factor mutated NSCLC, and in combination with chemo-radiation therapy in patients with unresectable stage III NSCLC. For the first study, the vaccine treatment was well tolerated, with observations of only some inject site reactions and flu-like symptoms. Increased antigen-specific immune response was observed in majority of the patients (84%). Antigen specific antibody and T cells are both increased, supporting further investigation of mRNA-based therapy with check-point inhibitors in treating NSCLC [5]. Moreover, Immunomic Therapeutics is collaborating with Dr. Duane Mitchell at the University of Florida on a Phase II proof of concept study using a pp65-lysosomal-associated membrane protein (LAMP)-based mRNA DC vaccine to treat patients bearing glioblastoma. pp65 is a major cytomegalovirus (CMV) protein that provides exceptional tumor specificity for glioblastoma and is designed to stimulate pp65-specific CD4^+^ and CD8^+^ T cell response. The previous phase I study showed a median overall survival of 35 months and progression-free survival of 31 months [143].

### mRNA vaccine encoding Neoantigen, personalized vaccine

Several obstacles limit the further application of TAA vaccines, including: (1) only limited TAAs have been identified for certain solid tumors resulting in limits of applications, and (2) patients harboring extensive variability in TAAs that gives rise to evasion of immune effectors and generation of resistance, (3) TAAs are also present in normal tissues. Vaccines against TTAs could potentially initiate central and peripheral tolerance responses, lowering vaccination efficiency. Tumor-specific antigens, termed neoantigens, are now the core targets of mRNA vaccines. Neoantigens are derived from random somatic mutations in tumor cells and not present in normal cells. Neoantigens could be recognized by the host immune system as a “non-self” motif and thus are an appealing target for cancer vaccine [136].

The first step in developing a personalized neoantigen vaccine is to identify and confirm patient-specific immunogenic non-synonymous somatic mutations expressed in the tumor. A biopsy of tumor tissue is taken for whole-exome, RNA, or transcriptome sequencing. Non-synonymous somatic mutations in cancer, such as point mutations and insertion-deletions, could be identified by comparing the sequences of the tumor and matched healthy tissues. Next, mutations with the highest immunogenicity are screened, analyzed, and identified using major histocompatibility complex (MHC) class I epitope prediction algorithms. Ranked lists of candidate antigens are further confirmed based on in vitro binding assay results. Various types of variant mutations can be targeted by neoantigen based vaccine [136].

Multiple delivery strategies have been developed for neoantigens, including synthetic long peptides (SLPs) [144] and nucleic acid (DNA/mRNA) based vaccines [145], either through direct injection of unformulated antigens, DC-based autologous transfer, or biomaterial-based delivery system [11]. In a pioneered phase I clinical study, a selected pool of 20 SLPs were s.c. administered together with adjuvant polyICLC to 6 patients with advanced cutaneous melanoma. These SLPs were shown to induce both CD4^+^ T cells and CD8^+^ T cells response. Four of the six patients were cancer-free 25 months post-treatment, demonstrating the viability of neoantigen vaccination in anticancer treatment [146]. However, peptides have limited immunogenicity, rapid clearance, and different physical-chemical properties restricting their clinical applications. Most recently, Sahin et al. reported that immunizing advanced melanoma patients in a clinical study using IVT mRNA encoding neoantigens through intranodal (i.n.) injection. The ultrasound-guided injection could maximize the capture of antigens by APCs. Potent T cell responses against multiple neoantigens were achieved in all the patients after vaccination [147]. Despite the encouraging initial results, the wide application of i.n. injection may be limited by the viability of the techniques and the difficulties for repeated dosing.

Non-viral platforms have until recently been applied to the delivery of mRNA encoding neoantigens. Multiple clinical trials investigating the safety and efficacy of mRNA vaccine encoding neoantigens are ongoing (Table 3). Moderna and Merck collaborated to develop mRNA-5671, a Kras personalized vaccine (encoding KRAS neoantigens), alone or in together with Merck’s PD-1 specific antibody KEYTRUDA (Pembrolizumab) to treat patients with pancreatic cancer in Phase I Trial [145]. LNPs were utilized to deliver mRNA-5671 intramuscularly every 3 weeks, 9 cycles in total. Results suggested that anti-tumoral immune response was developed and the formulation is overall well-tolerated. Another product is mRNA-4157, a personalized vaccine encapsulated in LNPs, for treating patients with resected solid tumors including melanoma, bladder carcinoma and NSCLC, as monotherapy or in combination with pembrolizumab (NCT03313778). The mRNA-4157 based mono and combination therapy with pembrolizumab showed an acceptable safety profile along with remarkable neoantigen-specific T cell responses. Twelve out of thirteen patients treated by monotherapy were reported to be disease-free [148]. BioNtech collaborated with Genentech to join the campaign and to evaluate the safety and efficacy of mRNA personalized vaccine, RO7198457 delivered by Lipo-MERIT platform in multiple phase I and II clinical trials.

## Conclusion and future perspectives

With the recent approval of two mRNA LNP vaccines to prevent COVID-19, mRNA vaccines are experiencing a considerable burst in preclinical and clinical research in both cancer and infectious disease fields. The challenges of developing cancer vaccines versus infectious disease vaccines lie in: firstly, most infectious disease vaccines are prophylactic, whereas cancer vaccines are therapeutic. The cases for preventive cancer vaccines are rare with only two FDA approved such vaccines, and these two vaccines are applied to prevent virus-induced malignancies (HPV and HBV) [3]. Though anti-cancer prophylactic vaccines are still under pre-clinical investigation, the clinical translation is limited by the difficulties of antigen predictions and the suboptimal immunogenicity. Secondly, most antigens for infectious disease (bacterial or virus-driven) are exogeneous motifs typically presented by the MHC_II_ molecule. Vaccines targeting these exogenous antig ens induce neutralizing antibodies mediated humoral response. In some cases, CD4^+^ T cell-mediated immune response is partially involved and required, whereas CD8^+^ cytotoxic T cells play crucial roles in the clearance of malignant cells with somatic mutations. Thus the anticancer therapeutic vaccine not only needs to boost humoral response, CD4+ T cell response but also needs to activate the MHC_I_ mediated CD^+^ 8 T cells responses, which further adds to the difficulties for efficient boosting of a robust antitumor immunity. Another major hurdle for efficient anticancer vaccine development is to identify and efficiently deliver highly immunogenic tumor-specific antigens. Tumor antigens are highly variable across different individuals, and some are less immunogenic and can invade the recognition by the host immune system. Even if the antigen is immunogenic, a suppressive microenvironment could prevent effective T cells’ infiltration and cause T cell exhaustion. Lastly, as a therapeutic vaccine for treating a chronic disease like cancer, multiple/repeatable dosing with higher dosage than prophylactic vaccines is required, raising the safety criteria for both mRNAs and the carriers.

Among other cancer vaccines, including DC-based vaccines and protein-based vaccines, mRNA stands out for several reasons: (1) mRNA could simultaneously encode multiple antigens, or a full protein with both MHC_I_ and MHC_II_ binding epitopes to facilitate both humoral and cellular adaptive immune response, providing a more intensified anti-tumor immunity. (2) Compared with DNA vaccine, mRNA vaccines are non-integrating, highly degradable, with no insertional mutagenesis potentials. Compared to protein or cell-mediated vaccines, the IVT production of mRNA is free of cellular and pathogenic viral components, with no infectious possibilities. Most mRNA vaccines tested in ongoing clinical trials are generally well tolerated, with rare cases of injection site reactions [7]. Systemic inflammation may be a major concern for mRNA vaccines due to its intrinsic immunostimulant-like function to activate the TLR7/8 pathway and to induce the type I IFN responses. However, type I IFN mediated innate immune response could be reduced by removal of the dsRNA contaminants, codon optimizations, and nucleotide modifications. The innate immune response could also be restricted to the local injection site by properly designing the delivery systems and changing the administration routes. The activation of type I interferon responses is not only associated with inflammation but also potentially with autoimmunity. Therefore, identifying individuals at an increased risk of autoimmune reactions before mRNA vaccination is another precautious step necessary to be taken [11]. (3) Another advantage of mRNA cancer vaccine is the rapid and scalable manufacturing. The mature manufacturing process of mRNA and formulation platform allows productions of a same or a new type of vaccine within a very short period.

Although identifying immunogenic TAAs/TSAs and overcoming suppressive tumor microenvironment still remain major hurdles for mRNA vaccine, the recent discovery and identification of neoantigens facilitate personalized vaccine treatment applications. mRNA encoded neoantigens have become the frontrunner in the personalized vaccine campaign. Multiple clinical studies led by the mRNA LNP pioneers BioNTech and Moderna, already presented promising results (with a readout of antitumor immunity) using personalized vaccines in several clinical trials treating multiple solid tumors, including metastatic melanoma and aggressive pancreatic cancers, opening a new era for therapeutic cancer vaccines. To further improve the potency of mRNA anticancer vaccines, multiple clinical trials are ongoing to evaluate the combination of mRNA vaccines with either cytokine therapies or checkpoint inhibitor therapies.

In conclusion, mRNA is a powerful and versatile cancer vaccine platform. Its successful development towards clinical translation will remarkably strengthen our ability to combat cancers. Future investigations should continue focusing on (but not limited to) understanding and utilizing the paradoxical inherent innate immunity of mRNA, improving the efficiency of antigen expression and presentation by designing advanced and tolerable delivery systems, and modifying mRNA structures to achieve extended and controlled duration of expression.

## Data Availability

Not applicable.

## Abbreviations

APCs: Antigen-Presenting Cells
LNPs: Lipid Nanoparticles
FDA: Food and Drug Administration
CAR: Chimeric Antigen Receptor
TAA: Tumor-Associated Antigen
TSA: Tumor-Specific Antigen
HPV: Human Papillomavirus
HBV: Hepatitis B Virus
HLA: Human Leukocyte Antigen
mRNA: Messenger RNA
IVT: In Vitro Transcription
SAM: Self-Amplifying mRNA
COVID-19: Coronavirus Disease 2019
MHC: Major Histocompatibility Complex
ORF: Open Reading Frame
UTR: Untranslated Region
dsRNA: Double Stranded DNA
PAMPs: Pathogen-Associated Molecular Patterns
PRRs: Pattern Recognition Receptors
TLR: Toll-Like Receptor
MyD88: Myeloid Differentiation Marker 88
IFN: Interferon
RIG-I: Retinoic Acid-Inducible Gene-I
OAS: Oligoadenylate Synthetase
PKR: RNA-Dependent Protein Kinase
ssRNA: Single Stranded RNA
DC: Dendritic Cells
eIF-2: Eukaryotic Initiation Factor-2
IFNAR: Interferon-α/β Receptor
eIF4E: Eukaryotic Translation Initiation Factor 4E
VCE: Vaccinia virus Capping Enzyme
ARCA: Anti-Reverse Cap Analogs
IFIT: Interferon-Induced Proteins with Tetratricopeptide Repeats
PABP: Poly(A) binding protein
HPLC: High-Pressure Liquid Chromatography
FPLC: Fast Protein Liquid Chromatography
STING: Stimulator of Interferon Genes
CD40L: CD40 Ligand
VRP: Viral Replication Particles
PEI: Polyethylenimine
RSV: Respiratory Syncytial Virus
GM-CSF: Granulocyte-Macrophage Colony-Stimulating Factor
RVG: Rabies Virus Glycoprotein G
LNP: Lipid Nanoparticle
DOTAP: 1,2-dioleoyl-3-trimethylammonium-propane
DOPE: 1,2-dioleoyl-sn-glycero-3-phosphoethanolamine
DSPC: 1,2-distearoyl-sn-glycero-3-phosphocholine
PEG: Polyethylene Glycol
EPO: Erythropoietin
DOE: Design of Experiment
ApoE: Apolipoprotein E
MPn-CH: Mannose-Cholesterol Conjugates
EM: Electron Microscope
FISH: Fluorescence in situ Hybridization
MHF: Microfluidic Hydrodynamic Focusing
SHM: Staggered Herringbone Mixing
GMP: Good Manufacturing Practice
PAMAM: Polyamidoamine
OVA: Ovalbumin
PBAE: Poly (beta-amino) Esters
APE: Amino Polyesters
pABOL: Poly (CBA-co-4-amino-1-butanol)
CART: Charge-Altering Releasable Transports
CPP: Cell-Penetrating Peptides
CFTR: Cystic-Fibrosis Transmembrane Regulator
CNE: Cationic Emulsions
DOTMA: 1,2-di-O-octadecenyl-3-trimethylammonium propane
LCP: Lipid/Calcium/Phosphate
LPR: Lipid-Polymer-RNA Lipopolyplexes
NSCLC: Non-Small Cell Lung Cancers
caTLR4: Constitutively Active TLR4
LAMP: Lysosomal-Associated Membrane Protein
CMV: Cytomegalovirus
SLP: Synthetic Long Peptide
